# Effect of Crosslinking Agent Concentration on the Properties of Unmedicated Hydrogels ^†^

**DOI:** 10.3390/pharmaceutics7030305

**Published:** 2015-09-09

**Authors:** Rachel Shet Hui Wong, Mark Ashton, Kalliopi Dodou

**Affiliations:** Department of Pharmacy Health and Wellbeing, Faculty of Applied Sciences, University of Sunderland, Science Complex, Wharncliffe Street, SR1 3SD Sunderland, UK; E-Mails: rachel.wong@research.sunderland.ac.uk (R.S.H.W.); mark.ashton@sunderland.ac.uk (M.A.)

**Keywords:** poly(ethylene oxide) (PEO), pentaerythritol tetraacrylate (PETRA), gel, transdermal, ultraviolet radiation, scanning electron microscopy, tensile strength, viscoelasticity

## Abstract

Novel polyethylene oxide (PEO) hydrogel films were synthesized via UV crosslinking with varying concentrations of pentaerythritol tetra-acrylate (PETRA) as crosslinking agent. The aim was to study the effects of the crosslinking agent on the material properties of hydrogel films intended for dermatological applications. Fabricated film samples were characterized using swelling studies, scanning electron microscopy, tensile testing and rheometry. Films showed rapid swelling and high elasticity. The increase of PETRA concentration resulted in significant increase in the gel fraction and crosslinking density (ρ_c_), while causing a significant decrease in the equilibrium water content (EWC), average molecular weight between crosslinks (${\bar{M}}_{c}$), and mesh size (ζ) of films. From the scanning electron microscopy, cross-linked PEO hydrogel network appeared as cross-linked mesh-like structure with interconnected micropores. Rheological studies showed PEO films required a minimum of 2.5% *w*/*w* PETRA to form stable viscoelastic solid gels. Preliminary studies concluded that a minimum of 2.5% *w*/*w* PETRA is required to yield films with desirable properties for skin application.

## 1. Introduction

Hydrogels are materials mainly constructed by the three-dimensional crosslinking of hydrophilic polymers. Their unique properties, such as high water content, biocompatibility and flexibility, give rise to potential applications in topical or transdermal drug delivery. Following the marketing of the primary wound dressings based on polyethylene oxide (PEO), e.g., Vigilon^®^, this non-ionic, synthetic, hydrophilic polymer has also gained interest from researchers as a suitable platform to transport drugs topically or transdermally [1,2,3]. PEO is an FDA-approved material and it is ideal for topical and transdermal drug delivery due to little to no immunogenicity, absence of residues, sediments, or vaporous elements when applied onto skin [4].

**Figure 1:**
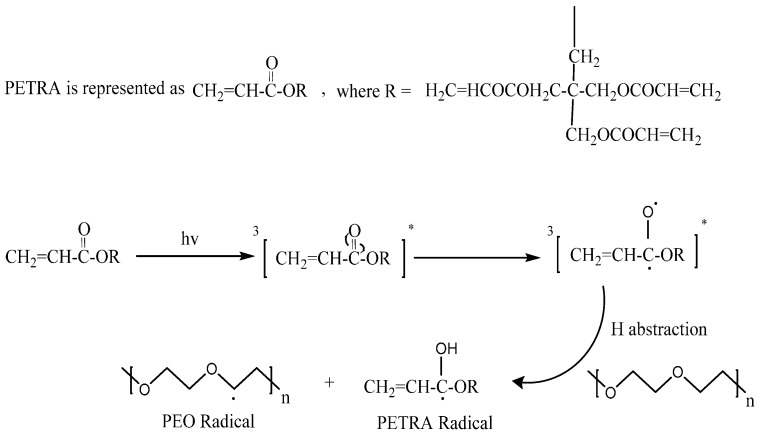
Generation of reactive PEO free radicals with the aid of PETRA as photoinitiator, figure adapted from Doytcheva *et al.* [6].

The crosslinking of PEO via gamma irradiation under room temperature has been well established since the early 1960s [5]. However, the expensive equipment and risk of working with ionizing radiation led to the adoption of ultraviolet (UV) initiated crosslinking method as alternative. Due to lack of photosensitive chromophoric groups in pure PEO, the addition of cross-linkable moieties, such as acrylate groups, is essential in order to achieve successful crosslinking of non-chromophoric PEO chains via UV irradiation [6]. The absorption of a photon causes acrylate groups to generate free radicals, and initiate the crosslinking process. It has been proposed that the radical photo-polymerisation of the acrylate groups of PETRA can result in a cross-linked network, which entraps the PEO chains. In addition, PEO free radicals are formed via photoinitiation by PETRA radicals [6]. The mechanism detailing the photoinitiation process resulting in the generation of PETRA and PEO radicals is illustrated in Figure 1. The major pathway by which crosslinks form, is through the recombination of two PEO radicals [6].

By adjusting the acrylate concentration, it is possible to control the degree of crosslinking of hydrogel films to achieve desirable material properties to allow them to qualify as patches for dermatological use.

The purpose of this work was to assess the impact of cross-linker on the material properties (*i.e.*, swelling, rheological and tensile properties, morphology) of unmedicated hydrogel films intended for skin application. To the best of our knowledge a detailed description of the mechanical properties of such films has not been reported before. PEO hydrogel film samples were synthesized via UV crosslinking with varying concentrations of pentaerythritol tetra-acrylate (PETRA) as cross-linking agent.

## 2. Experimental Section

### 2.1. Materials

Polyethylene oxide (PEO) ($\overset{=}{M}w$ = 1,000,000 g/mol) and pentaerythritol tetraacrylate (PETRA) were purchased from Sigma–Aldrich (Dorset, UK). All materials were used as received. Deionized water (Triple Red, Long Crendon, UK) was used for dissolving PEO and swelling of the PEO cross-linked films.

### 2.2. Sample Preparation

#### 2.2.1. Synthesis of PEO Hydrogel Films

Eight grams of PEO were premixed in distilled water (100 mL) under intensive stirring (IKA^®^ Werke GmbH & Co. KG, Staufen, Germany) for 72 h until a homogeneous gel like mixture was formed. A known amount of PETRA (1% *w*/*w*, 2.5% *w*/*w*, 5% *w*/*w*, and 10% *w*/*w*) was added into the premixed gel, and the entire mixtures were further stirred for 8 h until homogeneous. The viscous mixture was allowed to stand for 16 h to remove entrapped air bubbles before it was cast onto a glass tile. The cast gel was dried in a fume cupboard at room temperature (22 °C) for two days to form a film. The thickness of the dry hydrogel film was measured using a micrometer (Duratool, Taichung, Taiwan). The circular film was then cut into smaller rectangular pieces (3 cm × 5 cm).

#### 2.2.2. UV Irradiation of PEO Hydrogel Films

The irradiation process was carried out using a 150 W medium pressure mercury lamp (TQ 150 Heraeus Noblelight GmbH, Hanau, Germany, UV emission = 248–579 nm, λ_max_ = 366 nm), installed inside a quartz jacket within a cylindrical quartz tube. Each piece of film was individually fixed on the inner glass wall of the UV reactor, with approximately 4 cm directly below the head for perpendicular irradiation to achieve optimal light intensity. The total irradiation time was 11 min under a flow of nitrogen. The films were turned upside down after 5.5 min.

### 2.3. Measurements

#### 2.3.1. Swelling Behavior

Four replicas of pre-weighed dry hydrogel discs (10 mm diameter) from each sample batch were immersed in distilled water for 72 h at 25 °C. The swollen gel discs were periodically removed from water, blotted with filter paper, and weighed. The percentage swelling of each sample was calculated using Equation (1) [7].

(1) $$\text{Percentage}\;\text{Swelling}\;\left(\%\right)=\;\frac{M_{t}-M_{0}}{M_{0}}\;\times \;100\%$$

where *M_t_* is the weight of swollen gel at time *t*, and *M*_0_ is the initial weight of initial dry gel.

The equilibrium water content (EWC), reflecting the maximum amount of water absorbed by gel samples was calculated using Equation (2) [8].

(2) $$\text{EWC}\;\left(\%\right)=\;\frac{M_{s}-M_{0}}{M_{s}}\;\times \;100\text{\%}$$

where *M*_s_ is the weight of swollen gel at equilibrium.

At the end of the swelling experiment, the thickness of the swollen gel discs were measured and recorded. The samples were dried in a vacuum oven until constant weight values were attained and reweighed. The gel fraction was calculated using the following equation [9]:

(3) $$\text{Gel}\;\text{Fraction}\;\left(\%\right)=\;\frac{M^{\prime }}{M_{0}}\;\times \;100\%$$

where *M*^′^ refers to the weight of dried hydrogels after extraction of water.

*Determination of Average Molecular Weight between Cross-Links, Crosslinking Density, and Mesh Size.* The calculation of average molecular weight between cross-links (${\bar{M}}_{c}$), crosslinking density (ρ_c_), and mesh size (ξ) of all PEO hydrogel formulations was based on the equilibrium swelling theory, assuming Gaussian distribution of cross-linked polymer chains. Since the non-ionic gel samples were cross-linked in a dry state, the following equation suggested by Flory and Rehner was used to estimate ${\bar{M}}_{c}$ [10]:

(4) $$\frac{1}{{\bar{M}}_{c}}=\;\frac{2}{{\bar{M}}_{n}}-\;\frac{(\frac{\bar{\upsilon \;}}{V_{1}})[\text{ln}\left(1-V_{2,s}\right)+V_{2,s}+X_{1}{\left(V_{2,s}\right)}^{2}}{\left[{\left(V_{2,s}\right)}^{\frac{1}{3}}-\frac{V_{2,s}}{2}\right]}$$

where $\bar{M_{n}}$ is the number average molecular weight of the initial uncross-linked polymer in the absence of cross-linking agent, $\bar{\upsilon \;}$ is the specific volume of polymer (*i.e*., the reciprocal of polymer density, $\bar{\nu \;}$ = 0.833 cm^3^/g for PEO), *V*_1_ is the molar volume of water (18.1 cm^3^/mol), $V_{2,s}$ is the polymer volume fraction, and $X_{1}$ is the polymer-solvent interaction parameter (for water-PEO interaction, $X_{1}$ = 0.45 at ~25 °C [11].

The polymer volume fraction ($V_{2,s}$), was determined as follow [12]:

(5) $$V_{2,s}={\left[1+\;\frac{\rho_{p}}{\rho_{w}}\left(\frac{M_{s}}{M_{0}}\;-1\right)\right]}^{-1}$$

where $\frac{M_{s}}{M_{0}}$ is the weight swelling ratio of swollen hydrogel at equilibrium, ρ_p_ is the polymer density (1.2 g/cm^3^ for PEO) [12], and ρ_w_ is the solvent density (1.00 g/cm^3^ for water).

Subsequently, the crosslinking density (ρ_*c*_) was calculated using $\bar{M_{c}}$ [7,13]:

(6) $$\rho_{c}=\frac{1}{\bar{\nu \;}{\bar{M}}_{c}}$$

The theoretical mesh size (ξ) of hydrogel samples was estimated according to Equation (7) [14].

(7) $$\xi ={\left(V_{2,s\;}\right)}^{-\frac{1}{3}}{\left(\;\frac{2\;C_{n}{\bar{M}}_{c}}{M_{r}}\;\right)}^{\frac{1}{2}}\;l$$

where *C*_n_ is the polymer characteristic ratio (4.1 for PEO) [10], M_r_ is the molecular weight of the repeating units of the composed polymer (44 g/mol for PEO), and *l* is the average bond length along the polymer chain (1.54 $\dot{A}$ for PEO) [14].

#### 2.3.2. Scanning Electron Microscopy

The morphology of hydrogel samples containing different concentrations of PETRA was evaluated using a scanning electron microscope (SEM) (Hitachi, Tokyo, Japan) operated in high vacuum mode at an accelerating voltage of 5 kV. The hydrogel samples were initially swollen to equilibrium to reveal their final cross-linked network structure, transferred into liquid nitrogen for 10 min, and freeze-dried in a Christ ALPHA 2–4 LD device (SciQuip, Newtown, UK) under a vacuum of 0.1 Pa at −70 °C for 48 h to thoroughly remove the water. The freeze-dried hydrogel samples were put into liquid nitrogen for a sufficient length of time, fractured with a razor blade to expose the internal structures, and stuck onto the sample holder. All samples were sputter-coated with gold for 2 × 105 s before observation.

#### 2.3.3. Tensile Testing

Tensile strength and Young’s modulus of film samples were obtained with a Lloyd LS1 Material Tester (AMETEK Test and Calibration Instruments, Bognor Regis, UK) at room temperature (22 °C), using a 5.6 N load cell, 21 mm/min preload stress speed, and extension rate of 100 mm/min. Three replicas of dry hydrogel films were previously swollen to equilibrium in distilled water for 72 h at 25 °C and were cut into rectangular shapes, with a gauge length of 40 mm and width of 10 mm. The samples were clamped and subjected to tension until breakage.

#### 2.3.4. Measurement of Rheological Properties

The rheological properties of equilibrated PEO hydrogel films were determined with a Malvern Kinexus rotational rheometer (Malvern Instruments Ltd, Malvern, UK), equipped with a 20 mm diameter stainless steel parallel plate. Oscillatory rheological measurements were carried to measure the moduli of the films as a function of shear strain (Amplitude Sweep test) and as a function of frequency (Frequency Sweep test). The film sample was fixed between the upper parallel plate and stationary surface, with the gap size set according to individual swollen film thickness (320–470 μm). All tests were performed in triplicate at 25 ± 0.1 °C.

*Amplitude Sweep.* The linear viscoelastic region (LVR) of all samples was determined with an amplitude sweep at incremental shear strains (1 to 10^6^ Pa) and a fixed frequency of 1 Hz. Frequency sweep test was performed subsequently after identifying the appropriate stress and strain values, which were within the field of LVR.

*Frequency Sweep.* Frequency sweep measurements of film samples were carried out at decreasing oscillating frequencies from 100 to 0.1 rad/s Hz. The mean elastic modulus (G’) and viscous modulus (G’’) were plotted *vs.* frequency.

#### 2.3.5. Statistical Analysis

Statistical analysis was performed using SPSS 17.0 (SPSS UK Ltd., Woking, UK) to determine statistical differences. The swelling data, tensile test data and rheological data were analyzed by one-way analysis of variance (ANOVA). The subgroup means were compared by *post hoc* Scheffe’s test. Statistical significance for all tests was set at a probability of *p* < 0.05.

## 3. Results and Discussion

### 3.1. Effect of PETRA Content on the Swelling Properties

**Figure 2:**
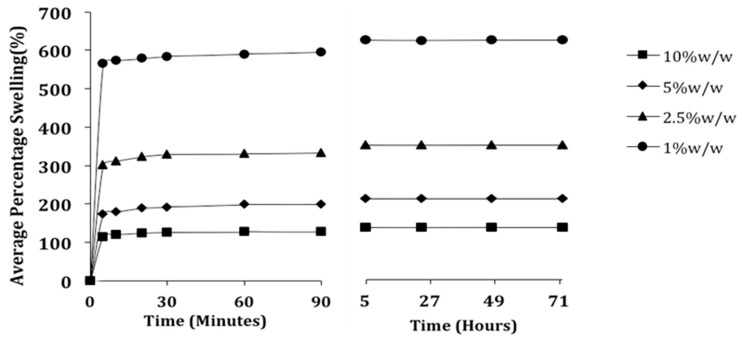
Effect of PETRA concentration on the % swelling values of PEO hydrogels.

The effect of PETRA concentration on the dependent parameters involved in the swelling experiments of PEO hydrogel samples was evaluated and is summarized in Table 1. The percentage swelling of hydrogel network structure is the most important parameter for swelling studies [15]. Figure 2 illustrates the effect of PETRA concentration on the percentage swelling of PEO hydrogel samples.

**Table 1:** Effect of PETRA concentration on the dependent variables involved in the swelling and rheological experiments of PEO hydrogel film samples. Values in brackets indicate the standard deviation from the reported mean.

PEO (*M*w = 1,000,000 g/mol)				
**Independent Parameter**				
PETRA Conc. (% *w*/*w*)	1	2.5	5	10
**Dependent Parameters**				
Thickness of dry film (µm)	210	200	200	230
Thickness of swollen film (µm)	470	350	320	320
Equilibrium swelling (%)	598.60 (±23.29) *	336.02 (±16.93) *	199.82 (±9.24) *	130.21 (±12.78) *
Equilibrium water content (%)	85.67 (±0.48) *	77.04 (±0.87) *	66.84 (±1.30) *	56.56 (±0.53) *
Gel fraction (%)	67.02 (±1.38) *	79.69 (±2.40) *	85.84 (±1.65) *	89.47 (±0.36) *
Average molecular weight between crosslinks, ${\bar{M}}_{c}$ (g/mol)	6643.65 (±516.21) *	2073.79 (±214.80) *	734.48 (±62.36) *	323.30 (±11.87) *
Crosslink density, ρ_c_ × 10^−4^ (mol/cm^3^)	1.79 (±0.15) *	5.835 (±0.57) *	16.45 (±1.56) *	38.39 (±2.71) *
Mesh size, ξ (nm)	10.72 (±0.41) *	4.98 (±0.08) *	2.73 (±0.08) *	1.64 (±0.04) *
Average elastic modulus within LVR, G’ (Pa × 10^5^)	–	1.23 (±0.34) ^a^	0.99 (±0.37) ^a^	1.12 (±0.37) ^a^
Average viscous modulus within LVR, G’’ (Pa × 10^4^)	–	1.80 (±0.83) ^a^	2.02 (±0.28) ^a^	2.19 (±0.50) ^a^

*n* = 4; ^a^ *n* = 3; * Significant differences between all groups; *p* < 0.05.

The % swelling of PEO hydrogels at all PETRA concentrations increased with time and reached equilibrium in the 5th hour. The higher concentrations of PETRA resulted in significantly lower swelling percentages due to the increase of crosslinks restricting the movement of polymer chains. This is further confirmed with the significant improvement in sample gel fraction (from 67.02% ± 1.38% to 89.47% ± 0.36%) by the addition of PETRA from 1% *w*/*w* to 10% *w*/*w*.

All EWC values (56.56% ± 0.53% – 85.67% ± 0.48%) calculated for the hydrogel samples used in this work were significantly higher than the water content of stratum corneum, which is normally 30% ± 5% [16]. This indicated the feasibility of our PEO hydrogel samples to be used as transdermal drug delivery patches, as the water inside the gel samples is high enough to act as natural penetration enhancer by simple hydration. The phenomenon is thought to change the packing order of stratum corneum, easing the diffusion of hydrophilic drug molecules into the skin [17].

The average molecular weight between cross-links (${\bar{M}}_{c}$), crosslinking density (ρ_*c*_), and the network mesh size (ξ) determined from equilibrium swelling experiments are key parameters in defining the microstructure of a cross-linked hydrogel network. ${\bar{M}}_{c}$ features the number average molecular weight of polymer chains between two adjacent crosslink junctions. This parameter allows the degree of crosslinking of hydrogel samples to be measured and expressed as ρ_c_. As expected, the increase of PETRA concentration led to significantly denser crosslinking of the gel network. This confirmed the role of PETRA as crosslinking agent. The mesh size (ξ) or sometimes referred as pore size, indicates the distance between two adjacent crosslinks. It is critical in controlling the drug diffusion rate as it reflects the amount of space available for a drug molecule to diffuse in or out of the swollen hydrogel network. Table 1 showed mesh size dependence on PETRA concentration. Gels prepared using 2.5% *w*/*w* PETRA concentration are classed as non-porous gels as their ξ values fall between 1 and 10 nm [18]. Interestingly, gels prepared using 1% *w*/*w* PETRA concentration exhibited different network properties as their ξ values fall within the typical range of a micro-porous gel, which is between 10 and 100 nm [18]. The ξ values of films prepared using 2.5% *w*/*w* and 5% *w*/*w* PETRA are comparable to those of polyethylene glycol (PEG)-containing hydrogels formulated for drug delivery via gamma irradiation [19]. In general, hydrogels with ξ values of around 1.5–7 nm do not cause disruption in the diffusion of small molecules [20].

### 3.2. Effect of PETRA Content on the Morphology Characteristics

**Figure 3:**
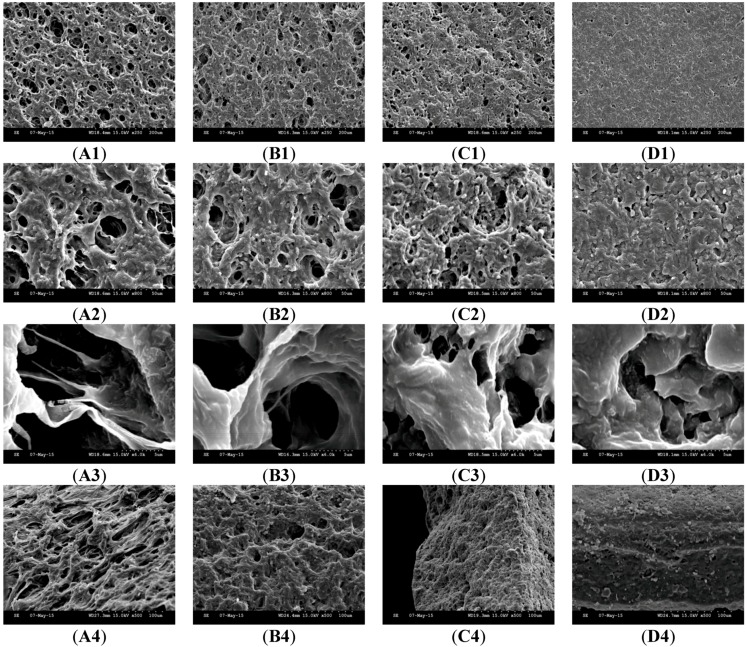

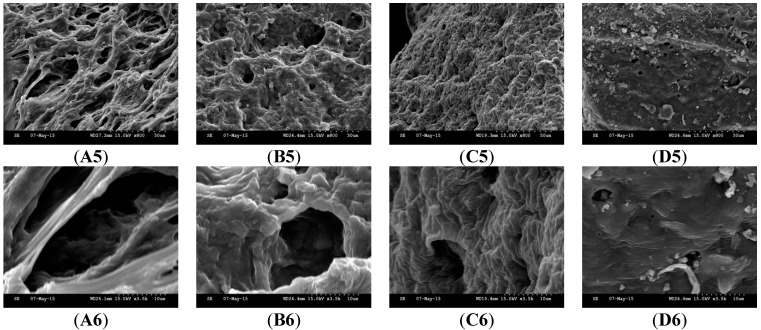
SEM images of freeze-dried PEO hydrogel network. (**A**) PEO-PETRA 1% *w*/*w*, (**B**) PEO-PETRA 2.5% *w*/*w*, (**C**) PEO-PETRA 5% *w*/*w*, (**D**) PEO-PETRA 10% *w*/*w*. Scale bars are 200, 50 and 5 μm for micrographs featuring the outer surface of hydrogel network (1–3), 100, 50 and 10 μm for micrographs featuring the cross section of hydrogel network (4–6).

The scanning electron microscopy (SEM) has been widely used to provide information regarding hydrogel’s surface topography and its characteristic network structure [21]. Figure 3 shows the scanning electron micrographs of freeze-dried PEO hydrogel network containing different PETRA concentration. The freeze-dried PEO hydrogel yielded from UV crosslinking with PETRA showed a cross-linked mesh-like structure, with interconnected micropores. Such a structure provides efficient channels for rapid water transport, which explains the capability of our hydrogel samples reaching equilibrium swelling in just 5 h (see Figure 2). The freeze-dried PEO hydrogel network was significantly denser with increasing PETRA concentration. The mesh sizes determined visually from the SEM micrographs were greater than those calculated from Equation (6). The calculated mesh sizes appeared to be more accurate as their values were estimated based on the equilibrium swelling experiments which is a much more in-depth analysis, accounting every part of the gel, while mesh sizes obtained from SEM micrographs are apparent sizes determined from one part of the gel. Nonetheless, the results from both methodologies explicitly indicated that the mesh size of PEO hydrogels decreased with the rise of PETRA concentration.

### 3.3. Effect of PETRA Content on the Mechanical Properties

For PEO hydrogel films to qualify for dermatological applications, they need to be flexible enough to follow the movement of the skin and sustain a comfortable feel, as well as possessing sufficient mechanical strength to resist abrasion caused by bodily movement or external objects (*i.e.*, clothing) [22]. This is especially true for applications on curved areas such as inside knees and elbows. Hence, high elongation (% elongation at break), high tensile strength, and elasticity (reflected through Young’s Modulus) are the desirable mechanical properties of films for dermatological applications. The tensile strength and percentage elongation at break of PEO hydrogel samples as a function of the PETRA concentration are shown in Figure 3.

**Figure 4:**
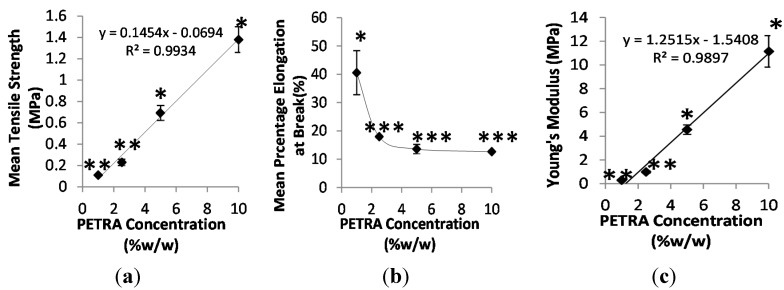
Effect of PETRA concentration on (**a**) tensile strength, (**b**) percentage elongation at break, and (**c**) Young’s modulus. *n* = 3; * significant differences between all groups; ** significantly different from PETRA concentrations = 5% and 10% *w*/*w*; *** significantly different from PETRA concentration = 1% *w*/*w*; *p* < 0.05.

It can be seen from Figure 4a that the tensile strength of PEO hydrogel samples increased linearly with increasing PETRA concentration due to the increased crosslinking density. Films containing 10% *w*/*w* PETRA concentration possessed highest tensile strength (1.41 ± 0.12 MPa), which is approximately 32 times higher than that of pure PEO films fabricated via electron beam (0.044 MPa) [23]. Statistical analysis showed that films prepared with 5% *w*/*w* and 10% *w*/*w* PETRA concentration possessed significantly higher tensile strength, as their obtained tensile values were significantly different between all tested groups (see Figure 4a). In terms of elongation properties, the increase of PETRA concentration caused the percentage elongation at break of gel samples to drop initially and plateau at 2.5% *w*/*w* PETRA. The increase of network crosslinking density generally decreases the film’s flexibility (percentage elongation at break) due to rigidity reasons. As expected, the Young’s moduli of hydrogel films increased linearly with increasing PETRA concentration. These moduli values are comparable to those of PEO/poly(ethylene glycol) diacrylate (PEGDA) hydrogels formulated for wound dressing application via electron beam [23]. Furthermore, the moduli of films containing 5% *w*/*w* and 10% *w*/*w* PETRA are within the literature Young’s modulus of the human skin (4.6–20 MPa) obtained from extension tests [24].

### 3.4. Effect of PETRA Content on the Rheological Properties

**Figure 5:**
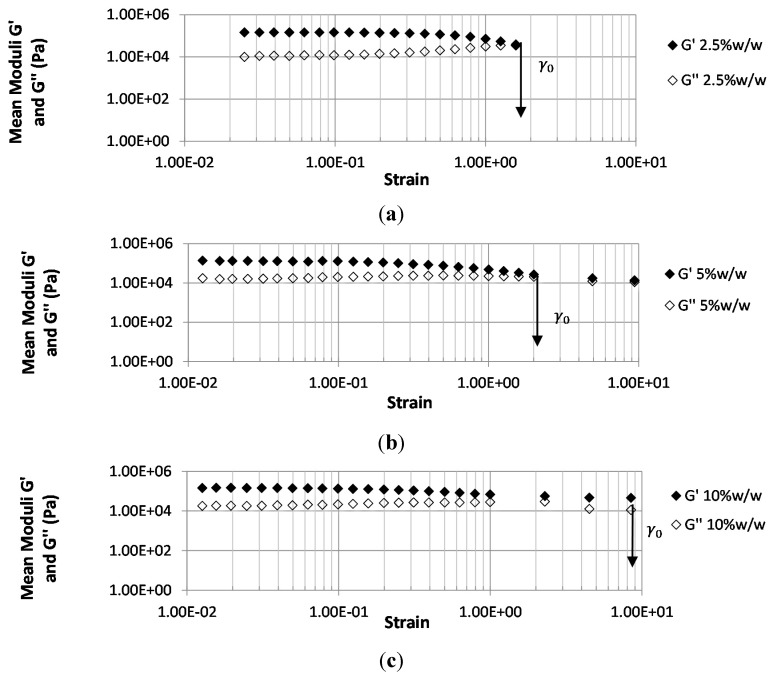
Log–log plot of shear moduli (elastic (G’) and viscous (G’’)) *vs.* strain for PEO hydrogels containing various PETRA concentrations (**a**) 2.5% *w*/*w*, (**b**) 5% *w*/*w*, and (**c**) 10% *w*/*w*. Frequency = 1 Hz and temperature = 25 °C.

Figure 5 indicates the effect of PETRA concentration on the linear viscoelastic regions (LVR) of hydrogel samples identified through the oscillatory stress sweep test. It can be seen from Figure 5 that PEO hydrogel films required at 2.5% *w*/*w* PETRA concentration to form stable, well-structured viscoelastic gels (presence of LVR). The average values of elastic modulus (G’) and viscous modulus (G’’) within the LVR obtained from all films prepared using different PETRA concentrations were tabulated in Table 1. No rheological values were obtained for films prepared with 1% *w*/*w* PETRA, as there was no LVR obtained during the rheological study. The absence of an LVR is indicative of incomplete crosslinking thus a non-viscoelastic and unstable structure. Moreover, the low mechanical strength of 1% PETRA films observed through tensile testing suggested that these films are not suitable for dermatological application. Rheological tests also revealed that both G’ and G’’ were not significantly influenced by the crosslinking density of the hydrogel network for films prepared using 2.5% *w*/*w* PETRA and above (see Table 1). This observation is in contrast to PEG-based hydrogels synthesized through thiol-ene photo-click chemistry, where increased crosslinking density had resulted in pronounced increment of G’ [25]. This could mean that through UV-crosslinking, and when within the mentioned PETRA concentration range, it is possible to modulate the mechanical and swelling profile of PEO hydrogels without compromising their rheological properties. Within the LVR, the viscoelasticity of hydrogel films was retained fully, as the polymer molecular arrangements are never far from equilibrium. The length of LVR and the magnitude of G’ within the LVR can be used as a measure for film structural stability, as structural properties are best related to elasticity [26]. PEO hydrogel films containing 2.5% *w*/*w*, 5% *w*/*w*, and 10% *w*/*w* PETRA concentration can withstand a strain up to a critical strain value (γ_0_) of 1.35, 2.00, and 8.46 respectively, without exhibiting changes in their elasticity. When the oscillatory strain amplitude (γ) is below the film’s critical strain (γ_0_), the structure of the hydrogel film is intact, and the material behaves in a solid-like manner due to G’ being greater than the G’’. Increasing the γ above the film’s γ_0_ indicates bond breakage [27] within the hydrogel network as the film behaves progressively towards a fluid-like manner with G’’ greater than G’, simultaneously, to a steep decrease in both moduli. Films containing 10% *w*/*w* PETRA concentration had the highest rheological stability and elasticity as their critical strain value was the highest.

**Figure 6:**
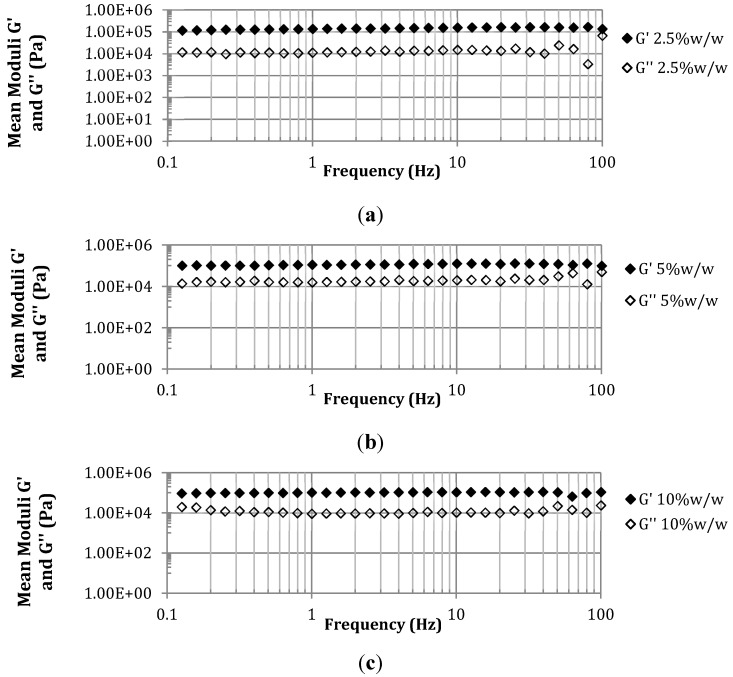
Log–log plot of average shear moduli (elastic (G’) and viscous (G’’)) *vs.* frequency for PEO hydrogel films containing various PETRA concentrations (**a**) 2.5% *w*/*w*, (**b**) 5% *w*/*w*, (**c**) 10% *w*/*w*. Strain (γ) = 0.1 and temperature = 25 °C.

The effect of PETRA concentration on the viscoelastic properties of PEO hydrogel films analyzed from the frequency sweep test is illustrated in Figure 6. The frequency sweep test was performed within the LVR, under constant strain (γ = 0.1). As seen from Figure 6, all tested films behaved as viscoelastic solids, with elastic modulus (G’) roughly one order of magnitude higher than viscous modulus (G’’) throughout the entire frequency range [28]. This observation is consistent with the solid-like elastic nature of hydrogel samples. The moduli of PEO hydrogel samples were fairly independent throughout the entire frequency range, showing no change in the degree of viscoelasticity. This is typical of gel behavior and indicative of a homogeneous cross-linked gel network.

## 4. Conclusions

PEO hydrogel films with rapid swelling and high elasticity were successfully fabricated through simple UV crosslinking with PETRA. The crosslinking density, governing the swelling, mechanical, and rheological properties of hydrogels can be easily tuned by varying the PETRA concentration. The macroscopic structure of the resulted films appeared as cross-linked mesh-like structure with interconnected micropores. From evaluation studies, PEO films containing PETRA concentration of 2.5% *w*/*w* and above have shown satisfactory mechanical and rheological properties. Next step to our studies is the preparation of medicated PEO hydrogel films.

## Symbols and Abbreviations

${\bar{M}}_{c}$: average molecular weight between crosslinks
ρ_c_: crosslinking density
ζ: mesh size
EWC: equilibrium water content
G’: elastic shear modulus
G’’: viscous shear modulus
LVR: linear viscoelastic region
PEO: polyethylene oxide
PETRA: pentaerythritol tetra-acrylate
UV: ultraviolet
